# Diagnostic accuracy of blood sucrose as a screening test for equine gastric ulcer syndrome (EGUS) in weanling foals

**DOI:** 10.1186/s13028-018-0377-5

**Published:** 2018-04-13

**Authors:** Michael Hewetson, Monica Venner, Jan Volquardsen, Ben William Sykes, Gayle Davina Hallowell, Ingrid Vervuert, Geoffrey Theodore Fosgate, Riitta-Mari Tulamo

**Affiliations:** 1 Department of Equine and Small Animal Medicine, Faculty of Veterinary Medicine, University of Helsinki, Helsinki, Finland; 2 Equine Clinique, Destedt, Germany; 3 School of Veterinary Sciences, University of Queensland, Brisbane, Australia; 4 School of Veterinary Medicine and Science, University of Nottingham, Nottingham, UK; 5 Institute of Animal Nutrition, Nutrition Diseases and Dietetics, Faculty of Veterinary Medicine, University of Leipzig, Leipzig, Germany; 6 Department of Production Animal Studies, Faculty of Veterinary Science, University of Pretoria, Pretoria, South Africa; 7 The Royal Veterinary College, Hawkshead Lane, North Mymms, Hertfordshire, AL9 7TA UK

**Keywords:** Bayesian, EGUS, EGGD, ESGD, Foal, Frequentist, Glandular, Permeability, Squamous, Sucrose, Ulcer, Weanling

## Abstract

**Background:**

Equine gastric ulcer syndrome is an important cause of morbidity in weanling foals. Many foals are asymptomatic, and the development of an inexpensive screening test to ensure an early diagnosis is desirable. The objective of this study was to determine the diagnostic accuracy of blood sucrose for diagnosis of EGUS in weanling foals.

**Results:**

45 foals were studied 7 days before and 14 days after weaning. The diagnostic accuracy of blood sucrose for diagnosis of gastric lesions (GL); glandular lesions (GDL); squamous lesions (SQL) and clinically significant gastric lesions (CSL) at 45 and 90 min after administration of 1 g/kg of sucrose via nasogastric intubation was assessed using ROC curves and calculating the AUC. For each lesion type, sucrose concentration in blood was compared to gastroscopy; and sensitivities (Se) and specificities (Sp) were calculated across a range of sucrose concentrations. Cut-off values were selected manually to optimize Se. Because of concerns over the validity of the gold standard, additional Se, Sp, and lesion prevalence data were subsequently estimated and compared using Bayesian latent class analysis. Using the frequentist approach, the prevalence of GL; GDL; SQL and CSL before weaning was 21; 9; 7 and 8% respectively; and increased to 98; 59; 97 and 82% respectively after weaning. At the selected cut-off, Se ranged from 84 to 95% and Sp ranged from 47 to 71%, depending upon the lesion type and time of sampling. In comparison, estimates of Se and Sp were consistently higher when using a Bayesian approach, with Se ranging from 81 to 97%; and Sp ranging from 77 to 97%, depending upon the lesion type and time of sampling.

**Conclusions:**

Blood sucrose is a sensitive test for detecting EGUS in weanling foals. Due to its poor specificity, it is not expected that the sucrose blood test will replace gastroscopy, however it may represent a clinically useful screening test to identify foals that may benefit from gastroscopy. Bayesian latent class analysis represents an alternative method to evaluate the diagnostic accuracy of the blood sucrose test in an attempt to avoid bias associated with the assumption that gastroscopy is a perfect test.

## Background

Equine gastric ulcer syndrome (EGUS) is an important cause of morbidity in foals, with a reported prevalence ranging from 22 to 57% [1–8]. Although it is most commonly recognized in older weanling foals, gastric ulceration has also been reported in neonatal foals as young as 24 h [2, 9, 10]. Ulcers have been reported in the squamous, glandular and duodenal epithelium, however the stratified squamous epithelium adjacent to the margo plicatus appears to be most commonly affected [2, 6]. Glandular and duodenal lesions are less common, but when present, they are more likely to be associated with clinical signs [6].

Four distinct clinical syndromes have been recognized: (1) asymptomatic or ‘silent’ ulcers, occurring most commonly in the squamous epithelium of the stomach along the margo plicatus; (2) symptomatic ulcers, which can occur in the squamous, glandular or duodenal epithelium, and which often manifest as ill thrift, inappetence, ptyalism, bruxism or colic; (3) pyloric or duodenal outflow obstruction with secondary ulceration of the gastric squamous epithelium and oesophagus, presumably due to reflux acid exposure; and (4) perforating ulcers that cause severe peritonitis and are invariably fatal [1, 3, 7, 11–14].

Considering the potentially fatal consequences of gastric ulceration in the foal and the fact that up to 57% of foals are asymptomatic, large scale screening on stud farms to ensure an early diagnosis and prompt treatment prior to the development of complications is desirable [7]. Currently, detection of EGUS by gastroscopy is considered to be the only reliable ante-mortem method for definitive diagnosis in foals, however it is unsuitable as a screening test, because it is expensive and time consuming.

Sucrose permeability testing represents a simple, economical alternative to gastroscopy for screening purposes, and the diagnostic accuracy of a blood sucrose test in adult horses has been reported [15]. Although the test was neither sensitive nor specific for detecting EGUS in adult horses with naturally occurring ulcers, direct extrapolation of the performance characteristics of the test from adults to foals is not possible, as fundamental changes in the gastric mucosal lining of the stomach that occur in the first 6 months of life may significantly alter epithelial permeability to sucrose when compared with adult horses [6, 16, 17]. Furthermore, the clinical syndromes demonstrated by foals with EGUS are different to that of adult horses, suggesting potential differences in the underlying pathophysiology that may be reflected as altered permeability to sucrose [13]. Concerns were also raised regarding the validity of the gold standard itself, and in particular the possibility that assessment via gastroscopy is under or overestimating the severity or depth of gastric lesions [18].

The objective of this study was therefore (1) to determine the diagnostic accuracy of blood sucrose as a potential screening test for EGUS in weanling foals by comparing it to gastroscopy as the gold standard, and (2) to investigate and compare the use of a Bayesian statistical approach that is based on the assumption that gastroscopy is an imperfect test i.e. estimation of sensitivity, specificity and disease prevalence when the true disease state is unknown [19].

## Methods

### Study design

The study was conducted as a blind comparison to a gold standard.

### Study population

Fourty-five weanling foals were included in the study. All foals were born and raised on the same stud farm; and were housed in covered barns with straw bedding and free access to concrete runs. Prior to weaning, mares and foals had access to grass hay and water ad libitum; and were fed approximately 13 kg per mare and foal per day of a total mixed ration comprising 3 kg corn silage, 6 kg grass silage, 2 kg oats, 0.5 kg straw, 0.3 kg soybean meal, and 50 g of a commercial mineral vitamin supplement. After weaning, foals were separated from their dams, and were fed the same total mixed ration or alfalfa chaff in addition to ad libitum grass hay.

Each foal was subjected to gastroscopy and sucrose permeability testing on two occasions; 7 days before and 14 days after weaning. Foals were randomly selected for gastroscopy based on the assumption that up to 20% of foals would be affected by naturally occurring gastric ulceration prior to weaning and 60% of foals would be affected post-weaning [2, 6]. Foals were excluded from the study if they had received omeprazole or non-steroidal anti-inflammatory drugs within 7 days prior to testing [20–22].

### Gastroscopy

Foals were fasted for 6 h prior to gastroscopy. Following completion of fasting, blood samples (10 mL) were collected in vacuumed clot tubes from the jugular vein, and foals were sedated with detomidine hydrochloride (Domosedan 10 mg/mL, OrionPharma, Finland) (0.02–0.04 mg/kg IV). Gastroscopy was subsequently performed using a previously described technique [23].

All endoscopic examinations were recorded and archived. For each foal, still-frame images were taken of the stomach from the right side of the stomach along the margo plicatus (MPRT), the dorsal part of the fundus, the greater curvature along the margo plicatus (MPGC), the lesser curvature along the margo plicatus (MPLC), the glandular mucosa in the region of the pylorus and the proximal duodenum [24].

### Administration of sucrose and collection of samples

Immediately following gastroscopy, 1 g/kg body weight (BW) of sucrose (Kidesokeri 530, Sucros Oy, Finland) was administered as a 10% solution via nasogastric tube to each foal. Serial blood samples (10 mL) were then collected in vacuumed clot tubes from the jugular vein at 45 and 90 min after administration of sucrose. Foals were not given access to food until the final blood sample had been collected. Foals that were not yet weaned were also prevented from suckling milk until the final blood sample had been collected, as it has been demonstrated that lactose may permeate across a damaged gastric mucosa, and will interfere with sucrose measurements due to reduced analytical specificity [25, 26]. Following blood collection, the serum was separated by centrifugation (10 min at 2000×*g*) and then stored in a freezer at − 80 °C until analysis.

### Lesion assessment

Following completion of data collection, still-frame images from each foal were reviewed independently by a board certified internist (BS) who was blinded to the results of the sucrose assay. For each set of images, the observer was asked to answer a series of dichotomous (yes or no) questions: does the foal have (1) gastric lesions? (2) glandular lesions? (3) squamous lesions? and (4) are the gastric lesions clinically significant? Clinically significant gastric lesions were used as a proxy indicator of ulcer severity and were defined as lesions that the observer would consider severe enough to warrant treatment. The term ‘lesion’ rather than ‘ulceration’ was used to enable the observer to report on the presence of other types of lesions (e.g. erosions or hyperaemia) in addition to ulceration.

### Inter-observer agreement

Observations for each foal were compared with observations made by two other board certified internists on the same set of still-frame images, and the level of agreement was calculated. These observers were also blinded to the results of the sucrose assay.

### Sample processing and analyses

Serum was analysed for sucrose using a previously validated gas chromatography–flame ionization detection (GC–FID) assay for quantifying sucrose in equine serum [25].

### Statistical analysis

All statistical analyses were interpreted at the 5% level of significance. The distributional form of quantitative data was assessed by calculating descriptive statistics, plotting histograms and performing the Anderson–Darling test using commercially available software (MINITAB Statistical Software, Release 13.32, Minitab Inc, State College, PA, USA). Correlation between the two sucrose concentration measurements was estimated using Spearman’s rho. The prevalence of gastric lesions identified using gastroscopy was estimated pre-weaning and post-weaning and formally compared using mixed-effects logistic regression that incorporated a random effect term to account for the repeated measures study design. For each lesion type, sucrose concentration in blood at 45 and 90 min was compared to gastroscopy as the gold standard; and sensitivities (Se), specificities (Sp), were calculated across a range of sucrose concentrations using mixed-effects logistic regression to account for the repeated observations on the same foals. All mixed effects regression models were implemented using commercially available software (IBM SPSS Statistics Version 23, International Business Machines Corp., Armonk, NY, USA).

The overall diagnostic accuracy of blood sucrose for diagnosis of GL; GDL; SQL; and CSL was assessed using receiver operator characteristics (ROC) curves and calculating the area under the curve (AUC). AUC was estimated using a bootstrap simulation approach that has been described previously but modified to account for the repeated measures study design [27, 28]. AUC of the two sucrose concentration measurements were statistically compared within the same bootstrap algorithm. Simulations were performed by writing Visual Basic code in a spreadsheet program (Excel, MS Office 2010, Microsoft Corporation, Redmond, WA, USA) and iterating using commercially available software (@Risk, Version 6.3.1, Palisade Corporation, Ithaca, NY, USA). The optimal cut-off for blood sucrose concentration was determined by calculating Youden’s index [29] and a modified version that weighted sensitivity twice as important as specificity (sensitivity * 1.33 + specificity * 0.67–1). Sensitivity was optimized to provide a practical threshold for screening purposes.

Sensitivity, specificity, and lesion prevalence were subsequently estimated using a Bayesian latent class (LC) model that was based on the assumption that gastroscopy is an imperfect test. The model is based on the Hui-Walter paradigm but modified for a Bayesian analysis [19, 30]. The model included adjustment for conditional dependence [31] between the two sucrose concentration tests and similar diagnostic test models have been described in more detail elsewhere [32, 33]. The base model was a three test (sucrose at 45 min, sucrose at 90 min, gastroscopy) and two population (pre-weaning, post-weaning) model that included adjustment for conditional dependence in sensitivity and specificity estimates for the two sucrose concentration assays. Sucrose concentration results were dichotomized into positive and negative based on the optimal cut-off identified using Youden’s index in the analysis that assumed gastroscopy was a perfect reference test. Diffuse, mildly informative, prior probability distributions (Table 1) were elicited based on published literature and expert opinion of the authors [2, 18]. Non-informative priors were used for the sucrose concentrations assay since prior information was lacking at the time of the study. Markov chain Monte Carlo (MCMC) techniques were implemented in available statistical software (WinBUGS Version 1.4, MRC Biostatistics Unit, Cambridge, UK). Iterate values of the MCMC process were expected to be highly correlated and only every 10th iterate was retained to alleviate this concern. Convergence was assessed by evaluating plots of model parameter iterates and by calculating the Gelman–Rubin statistic [34]. The first 200,000 iterations were discarded as the burn-in and inferences were made based on the subsequent 40,000. Median values were used as point estimates and 95% probability intervals (PI) were calculated as the 2.5th–97.5th percentiles of the posterior distributions.

**Table 1:** Beta prior probability distributions used in the Bayesian latent class analysis to estimate sensitivity and specificity of test to identify gastric ulcers in foals

Population and tests	Measure	Prior probability distribution (β)	Mean	Median	90% probability interval
Pre-weaning	Prevalence	2, 8	0.20	0.18	0.041, 0.388
Post-weaning	Prevalence	6, 4	0.60	0.61	0.345, 0.831
Endoscopy	Sensitivity	8, 2	0.80	0.82	0.571, 0.959
	Specificity	99, 1	0.99	0.99	0.970, 0.999
Sucrose 45	Sensitivity	1, 1	0.50	0.50	0.025, 0.975
	Specificity	1, 1	0.50	0.50	0.025, 0.975
Sucrose 90	Sensitivity	1, 1	0.50	0.50	0.025, 0.975
	Specificity	1, 1	0.50	0.50	0.025, 0.975

Inter-observer agreement was summarised as the percentage of perfect (100%) agreements between observers for each diagnostic criterion and the Kappa coefficient (K) was calculated.

## Results

### Foals

A total of 90 data sets were collected, comprising 45 weanling foals that were subjected to gastroscopy and sucrose permeability testing on 2 occasions: 7 days before and 14 days after weaning. There were 26 colts and 19 fillies. At the time of testing, foals ranged from 182 to 200 days of age (median, 190 days). Body weight ranged from 207 to 303 kg (median, 253 kg). None of the foals in this study had been reported to be demonstrating clinical signs consistent with gastric ulceration at the time of gastroscopy.

### Gastroscopy

The prevalence of gastric lesions in foals prior to weaning was 21%; with a similar proportions of foals affected by squamous lesions (7%) and glandular lesions (9%) (Table 2). In comparison, the prevalence of gastric lesions in foals after weaning was 98%, with a predominance of squamous lesions (97%) over glandular lesions (59%) (Table 2). The incidence rate over the period between samplings was 30% new cases with gastric lesions per foal week.

**Table 2:** Prevalence of gastric lesions identified via endoscopy in 45 weanling foals

Lesion type	Pre-weaning		Post-weaning	
	Gold standard^a^	Bayesian LC^b^	Gold standard^a^	Bayesian LC^b^
	Prevalence % (95% CI)	Prevalence % (95% PI)	Prevalence % (95% CI)	Prevalence % (95% PI)
GL	21 (9, 42)	42 (29, 57)	98 (93, 100)	92 (83, 98)
GDL	9 (4, 20)	36 (23, 51)	59 (40, 76)	88 (77, 95)
SQL	7 (3, 19)	36 (21, 50)	97 (89, 99)	92 (83, 98)
CSL	8 (3, 19)	37 (24, 51)	82 (65, 92)	91 (81, 97)

*LC* latent class, *CI* confidence interval, *PI* probability interval; Bayesian analog of the confidence interval. *GL* gastric lesion, *GDL* glandular lesion, *SQL* squamous lesion, *CSL* clinically significant lesion

^a^Calculated relative to direct observation of lesions via endoscopy as the gold standard but adjusted for repeated measurements using mixed-effects logistic regression

^b^Based on Bayesian latent class analysis with sucrose tests evaluated at the 24 µmol/L cut-off and endoscopy assumed to be an imperfect test

**Fig. 1:**
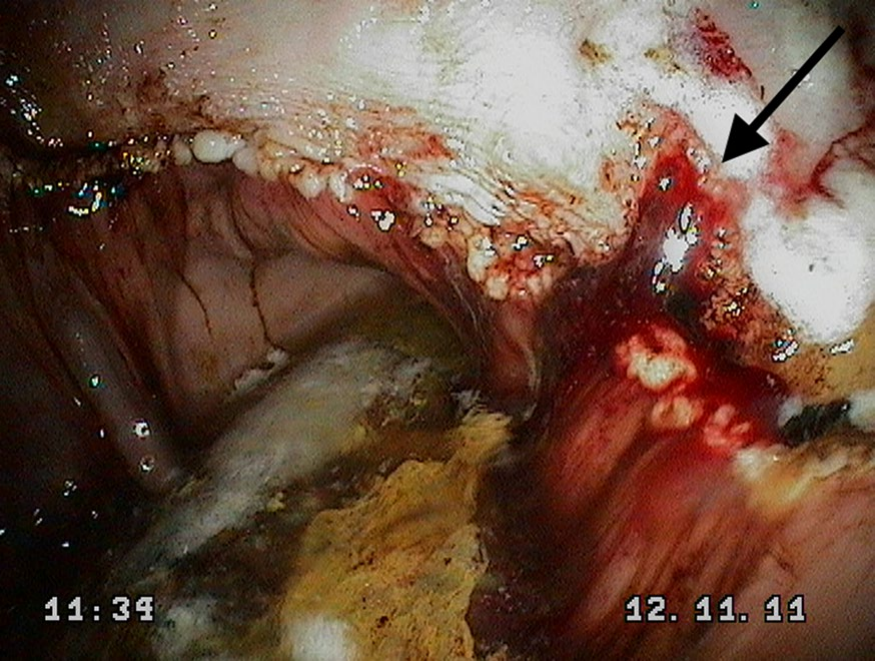
Clinically significant gastric lesions in a foal 14 days after weaning characterised by deep ulceration in the squamous epithelium and acute haemorrhage (black arrow). The image was obtained from the lesser curvature of the stomach along the margo plicatus. Blood sucrose concentration at 45 and 90 min for this foal was 35.4 and 34.3 µmol/L respectively. This foal would have been correctly identified as positive for EGUS using the blood sucrose test

Squamous lesions were most frequently observed in the region of the cardia and along the lesser curvature of the stomach adjacent to the margo plicatus. The lesions varied from large focal ulcers to extensive lesions with areas of apparent deep ulceration and active haemorrhage (Fig. 1). Glandular lesions were most frequently observed around the pylorus and consisted of focal or multifocal flat haemorrhagic or fibrinous lesions, often surrounded by a region of intense hyperaemia. Ulcer severity varied from mild to severe (Fig. 1), with a marked increase in the prevalence of clinically significant lesions following weaning (Table 2).

### Sucrose permeability

**Fig. 2:**
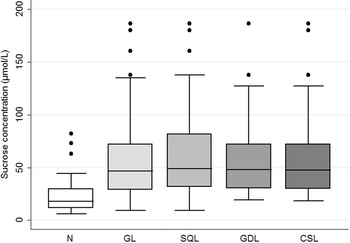
Scatter plot of sucrose concentrations measured at 45 and 90 min post sucrose administration. There was a strong positive correlation between sucrose concentrations at both time points (ρ = 0.935; P < 0.001)

All foals tolerated sucrose permeability testing and no adverse effects were noted following administration of the sucrose solution. On analysis of the serum samples pre- and post-weaning, all foals demonstrated an increase in sucrose concentration over time following oral administration of sucrose, and there was a strong positive correlation between sucrose concentrations at 45 and 90 min (Fig. 2).

**Fig. 3:**
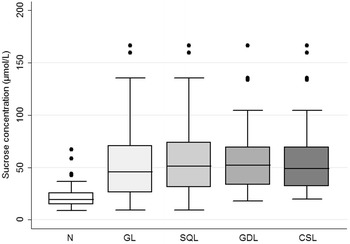
Gastric sucrose permeability: Box and whisker plot of blood sucrose concentrations from normal weanling foals (n = 34); and foals with gastric lesions (n = 56), glandular lesions (n = 32), squamous lesions (n = 48) or clinically significant lesions (n = 40) at 45 min after administration of 1 g/kg of sucrose via nasogastric intubation. *N* normal, *GL* gastric lesions, *GDL* glandular lesions, *SQL* squamous lesions, *CSL* clinically significant lesions

**Fig. 4:**
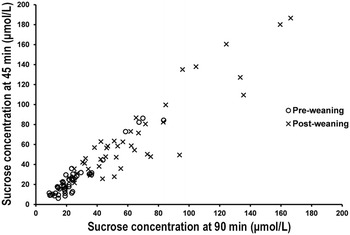
Gastric sucrose permeability: Box and whisker plot of blood sucrose concentrations from normal weanling foals (n = 34); and foals with gastric lesions (n = 56), glandular lesions (n = 32), squamous lesions (n = 48) or clinically significant lesions (n = 40) at 90 min after administration of 1 g/kg of sucrose via nasogastric intubation. *N* normal, *GL* gastric lesions, *GDL* glandular lesions, *SQL* squamous lesions, *CSL* clinically significant lesions

Of the 90 data sets comprising 45 weanling foals that were subjected to gastroscopy and sucrose permeability testing pre- and post-weaning, the mean ± SD serum sucrose concentration at 45 min was 23.99 ± 17.91 µmol/L for normal foals (n = 34); 57.56 ± 41.30 µmol/L for foals with GL (n = 56); 59.24 ± 38.15 µmol/L for foals with GDL (n = 32); 62.12 ± 42.73 µmol/L for horses with SQL (n = 48); and 59.44 ± 40.55 µmol/L for foals with CSL (n = 40) (Fig. 3). The mean ± SD serum sucrose concentration at 90 min was 23.83 ± 13.26 µmol/L for normal foals (n = 34); 54.84 ± 36.58 µmol/L for foals with GL (n = 56); 58.01 ± 35.19 µmol/L for foals with GDL (n = 32); 59.08 ± 37.51 µmol/L for foals with SQL (n = 48); and 58.52 ± 37.31 µmol/L for foals with CSL (n = 40) (Fig. 4).

### Diagnostic accuracy of blood sucrose for diagnosis of EGUS

**Fig. 5:**
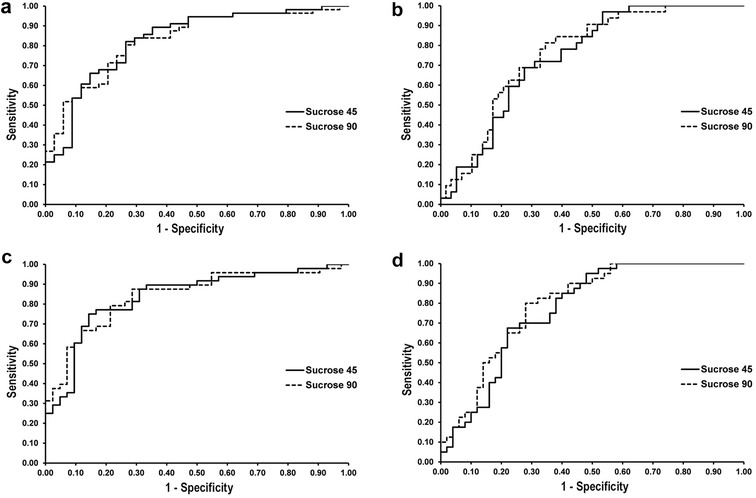
**a** Receiver-operating characteristic (ROC) curves depicting the ability of sucrose concentration to predict the presence of gastric lesions (**a**); glandular lesions (**b**); squamous lesions (**c**) and clinically significant gastric lesions (**d**) in weanling foals at 45 and 90 min after administration of 1 g/kg of sucrose via nasogastric intubation

ROC curves and the area under the curve (AUC) for each diagnostic criterion at 45 and 90 min after sucrose administration are illustrated in Fig. 5.

A sucrose concentration of 24 µmol/L was selected as the optimal cut-off for discriminating between normal foals and foals with EGUS. This was done to improve the practicality of the test for field purposes. Using the selected cut-off value, the sensitivity and specificity of blood sucrose at 45 and 90 min for diagnosis of GL; GDL; SQL; and CSL was calculated using frequentist estimation. Additional Se, Sp, and lesion prevalence data were estimated using Bayesian latent class analysis for the purposes of comparison (Table 3). The Bayesian model also estimated Se and Sp dependence terms for the sucrose tests (data not presented).

**Table 3:** Diagnostic accuracy of blood sucrose for diagnosis of EGUS using traditional and Bayesian latent class analyses in 45 foals evaluated pre and post weaning

Lesion type	Test	Parameter	Gold standard^a^	Bayesian LC^b^
			Estimate % (95% CI)	Estimate % (95% PI)
GL	Sucrose 45	Sensitivity	89 (78, 95)	89 (77, 97)
		Specificity	65 (47, 79)	83 (65, 95)
	Sucrose 90	Sensitivity	84 (72, 92)	81 (69, 91)
		Specificity	71 (53, 84)	95 (80, 100)
	Endoscopy	Sensitivity	NA	81 (70, 90)
		Specificity	NA	99 (95, 100)
GDL	Sucrose 45	Sensitivity	95 (79, 99)	97 (90, 100)
		Specificity	47 (34, 60)	87 (69, 98)
	Sucrose 90	Sensitivity	90 (73, 97)	91 (80, 99)
		Specificity	55 (42, 67)	97 (83, 100)
	Endoscopy	Sensitivity	NA	47 (36, 59)
		Specificity	NA	99 (95, 100)
SQL	Sucrose 45	Sensitivity	89 (77, 96)	90 (78, 98)
		Specificity	55 (39, 70)	77 (55, 91)
	Sucrose 90	Sensitivity	87 (74, 94)	84 (72, 94)
		Specificity	64 (48, 78)	94 (76, 100)
	Endoscopy	Sensitivity	NA	75 (62, 87)
		Specificity	NA	99 (94, 100)
CSL	Sucrose 45	Sensitivity	94 (80, 98)	95 (88, 99)
		Specificity	52 (38, 66)	89 (71, 99)
	Sucrose 90	Sensitivity	90 (75, 96)	89 (79, 97)
		Specificity	58 (44, 71)	98 (85, 100)
	Endoscopy	Sensitivity	NA	60 (48, 71)
		Specificity	NA	99 (96, 100)

*CI* confidence interval, *PI* probability interval; Bayesian analog of the confidence interval. *NA* not able to calculate since endoscopy is considered the gold standard reference test

*GL* gastric lesion, *GDL* glandular lesion, *SQL* squamous lesion, *CSL* clinically significant lesion

^a^Calculated relative to direct observation of lesions via endoscopy as the gold standard

^b^Based on Bayesian latent class analysis with sucrose tests evaluated at the 24 µmol/L cutoff and endoscopy assumed to be an imperfect test

### Inter-observer agreement

When asked to answer if each foal has (1) gastric lesions; (2) glandular lesions; (3) squamous lesions; and (4) are the gastric lesions clinically significant?; perfect agreement between-observers within the 90 sets of observations was achieved on average, in 62% (K = 0.46; P < 0.0001; 95% CI 0.28–0.64); 52% (K = 0.42; P < 0.0001; 95% CI 0.30–0.54); 68% (K = 0.67; P < 0.0001; 95% CI 0.55–0.79); and 75% (K = 0.56; P < 0.0001; 95% CI 0.29–0.54) of the cases respectively.

## Discussion

The objective of this study was to evaluate the sucrose blood test as a screening test for EGUS in foals by determining its performance characteristics in a group of foals pre- and post-weaning. ROC curve analysis was used as a graphical representation of the cut-off dependency of the test across a range of sucrose concentrations; with the AUC representing the overall diagnostic accuracy of the test. The AUC ranged from 0.75 to 0.85 depending upon the lesion type and time of sampling, indicating that blood sucrose concentration effectively discriminates between normal foals and foals with (1) gastric lesions; (2) glandular lesions; (3) squamous lesions; and (4) clinically significant lesions at 45 and 90 min after administration of sucrose, and is therefore considered to be a moderately accurate test [35].

A cut-off value was inserted on the continuous scale of test results that allowed calculation of Se and Sp for foals with EGUS. In the case of weanling foals, the prevalence of EGUS is high [7], and missing any diseased animal has potentially serious consequences [3, 11, 12], therefore a cut-off value towards the upper part of the ROC curve that maximised Se was selected. Using the selected cut-off, the Se of the blood sucrose test for detecting EGUS was high (84–95%), irrespective of whether the sample was taken at 45 or 90 min after sucrose administration (Table 3). This allows for a certain amount of leeway for the practitioner to collect the sample within 90 min but no less than 45 min after administration of the sucrose, thus improving the practicality of the test. A high Se is ideal for a screening test as it correctly identifies most foals with gastric ulcers, remembering that many weanling foals do not show clinical signs of gastric ulceration, even in the face of severe disease [7]. Another way of looking at this is that a negative result is a very reliable way to rule out gastric ulcers, as the blood sucrose test rarely misses foals with gastric ulcers. The Sp is poor however (47–71%), which means that a positive test is not a very good way of correctly excluding foals without gastric ulcers, as there is a high false positive rate. In the context of how the test is intended to be used (i.e. as a screening test), this is of no major consequence however, as the risk of a foal without gastric lesions being incorrectly classified as positive for gastric ulceration and undergoing an unnecessary confirmatory gastroscopy far outweighs the risk of incorrectly classifying a foal with a gastric ulcer as normal, and dealing with the potentially fatal and far more expensive consequences of a pyloric stricture or perforating ulcer.

It is in fact not unusual for non-invasive permeability tests to be characterised by a high sensitivity and a low specificity for the very reasons outlined above; a good example being the lactulose/mannitol excretion ratio test which is currently used for non-invasive for screening against celiac disease in human patients [36]. Volgesang et al. [37] investigated the value of a variety of non-invasive tests used to screen for celiac disease by comparing them to an intestinal biopsy as the gold standard, and found that the urinary lactulose/mannitol excretion ratios had a sensitivity of 100% but a specificity of only 55%. Despite this, the test is still considered to be a very useful cost-effective screening tests to correctly identify patients that would benefit from more invasive diagnostic tests, including an intestinal biopsy.

In this study we aimed to determine the diagnostic accuracy of the sucrose blood test as a screening test for EGUS in weanling foals and therefore we selected foals that were approximately 6 months of age at the time of testing, and tested them on two occasions; 7 days before and 14 days after weaning. The prevalence of gastric lesions prior to weaning was 21% and increased to a staggering 98% within 2 weeks of weaning. This is the first study that has reported prevalence data for foals immediately after weaning despite a wealth of anecdotal evidence suggesting weaning as a risk factor for EGUS, and underscores the importance of gastric ulceration in this age group. A wide spectrum of disease was represented in the study population, ranging from normal to extensive bleeding lesions with areas of apparent deep ulceration characteristic of EGUS severity score 4 [38]. Despite the severity of disease in some foals, none demonstrated clinical symptoms at the time of testing, making the results of this study very relevant to the general population, where many foals with severe ulcers do not demonstrate clinical signs, and therefore the benefit of a sensitive screening test cannot be underestimated [7].

Sucrose is safe and non-toxic, and is therefore suitable as a permeability marker in foals [39]. Although specific studies on the safety of oral sucrose administration in weanling foals are not available, oral tolerance tests to investigate disaccharide digestion in neonatal foals have been performed, and at a dose of 1 g/kg BW as a 20% solution w/v, no deleterious effects were seen [40]. Oral administration of hyperosmolar solutions has the potential risk of causing an osmotic diarrhoea, however none of the foals in our study or the aforementioned study developed diarrhoea, suggesting that a dose of 1 g/kg BW as a 10 or 20% solution w/v is well tolerated.

In a previous study, it was demonstrated that lactose may interfere with sucrose during GC–FID analysis of serum [25]. This may present a problem when determining serum sucrose concentrations in unweaned foals, as milk contains high concentrations of lactose. Some of this lactose may permeate across a damaged gastric mucosa and accumulate in the serum, where it will interfere with sucrose measurements due to reduced analytical specificity [25, 26]. This problem can be solved by fasting unweaned foals for at least 6 h prior to sucrose testing to ensure adequate time for excretion of any milk derived lactose that may have permeated across the gastric mucosa [41].

This study focused on validating the sucrose permeability test for weanling foals. It is conceivable that the test would also be of value for screening neonatal foals for gastric ulcers. Rather than prophylactic treatment of hospitalized neonatal foals with acid suppressing drugs, which have been demonstrated to increase the odds of developing diarrhoea, and may in fact not actually reduce the incidence of gastric ulceration, perhaps a more reasonable approach would be to rule out ulcers using the non-invasive blood sucrose test, and only target treatment to those foals that are positive [42]. There are however, some important physiological differences in neonatal foals that may limit the usefulness of the test. While older foals have been shown to have normal sucrase activity in the small intestine, oral tolerance tests performed on neonatal foals between the ages of 1 and 5 days postpartum have demonstrated that foals of this age do not yet have the ability to digest sucrose [40]. This is likely to be related to age related changes in sucrose hydrolysis and monosaccharide absorption along the small intestine, with older animals showing an improved ability to hydrolyse sucrose and to absorb monosaccharides [43]. This will of course have significant implications for the sucrose permeability test, as it implies that in neonates, sucrose most likely passes through the gastrointestinal tract unaltered, and therefore has the potential to permeate through any diseased mucosa, irrespective of its anatomical location in the gastrointestinal tract. This means that in the neonatal foal, it is likely that the test is not specific for the stomach. From this point of view, sucrose permeability testing in neonatal foals may be a more useful test for generalized gastrointestinal disease and further studies are certainly warranted. For example, in man, increased intestinal permeability has been documented in premature infants compared to healthy term infants and non-invasive assessment of intestinal permeability using sugar tests has been demonstrated to be useful in monitoring the effects of experimental (nutritional) therapy in these patients, as they are thought to be predisposed to necrotising enterocolitis due to their enhanced intestinal permeability [44, 45].

When considering gastric permeability in the foal, changes in the gastric mucosal lining of the stomach (epithelial desquamation) that occurs in the first 6 months of life may alter epithelial permeability to sucrose when compared with adult horses [6, 16, 17]. In this study, the blood sucrose cut-off for discriminating between normal foals and foals with EGUS was approximately five times higher when compared with adult horses [15], suggesting that foals in this age group do in fact have increased gastric permeability, irrespective of their disease status. The reason for this is not immediately clear, but it is likely to be associated with age related changes in intestinal tight junction permeability that is independent of the increased permeability caused by erosion or ulceration, which is thought to occur as a direct result of gaps in the epithelium [26, 46, 47]. Alternatively, epithelial desquamation in this age group of foals may result in increased paracellular permeability through the formation of ‘extrusion zones’ left following removal of dead cells from the mucosal surface [48]. Irrespective of the underlying reason, this relative increase in permeability of the gastric mucosa in foals may be one explanation as to why the diagnostic accuracy of the sucrose blood test was better in this population group. It is interesting to note that a similar age related change in gastroduodenal permeability occurs in human patients, with a decline in the recovery of urinary sucrose reported in adults when compared to children [49]. Further in vitro investigations using endoscopic biopsies in Using chambers may help elucidate the reasons for this age related difference in gastric permeability in horses; and help guide future clinical research in the field of equine gastrointestinal permeability [50–53].

When considering gastric permeability in the foal, changes in the gastric mucosal lining of the stomach that occur in the first 6 months of life may alter epithelial permeability to sucrose when compared with adult horses [6, 16, 17].

The diagnostic accuracy of the blood sucrose test was determined by comparing it to gastroscopy as the gold standard, however the validity of the gold standard itself has been questioned [15]. In particular, it has been demonstrated that there is poor correlation between endoscopic assessment of gastric ulcers antemortem and histological appearance at necropsy [18]. This has the potential to significantly affect the diagnostic performance characteristics of the test, as gastroscopy may under or overestimate the severity or depth of gastric lesions, leading to an erroneous comparison with sucrose concentration in blood. To explore the implications of these limitations on the diagnostic performance characteristics of the test, a Bayesian statistical approach was used in addition to the traditional frequentist approach. Bayesian latent class models are important mathematical frameworks to study the prevalence and the performance of diagnostic tests in the absence of a gold standard test, and in this case, the model was used based on the assumption that gastroscopy is an imperfect test i.e. the true disease state in the population was assumed to be unknown. In a Bayesian analysis, data are combined with prior information that expresses expert opinions and other sources of knowledge. As such the model allows for the inclusion of prior probabilities to account for current knowledge that is subsequently combined with the information contained in the experimental data to determine posterior probabilities. The elicitation of an informative prior is a difficult and subjective process that requires a careful dialogue between the statistician and the expert [54]. In this study, informative priors were elicited based on published literature reporting the prevalence of EGUS in different age groups of foals, and the expert opinion of the authors [2, 6]. When compared to the frequentist approach, estimates of Se and Sp in this study were consistently higher when using a Bayesian approach, with Se ranging from 81 to 97%; and Sp ranging from 77 to 97%, depending upon the lesion type and time of sampling (Table 3).

## Conclusions

Blood sucrose is a useful screening test for detecting gastric ulcers in weanling foals as it fulfils all the major criteria for a screening test: it is (1) economical, so that a large proportion of the population can be tested at a relatively low cost; (2) minimally invasive and acceptable to owners; (3) easy to perform; and (4) accurate, with good sensitivity. Due to its poor specificity, it is not expected that the sucrose blood test would replace gastroscopy, however it may represent a clinically useful screening test that can be used to identify foals that may benefit from gastroscopy. The usefulness of this test for diagnosis of gastric ulceration in neonatal foals is yet to be determined. A Bayesian statistical approach was demonstrated in this study, and represents an alternative method to evaluate the diagnostic accuracy of the blood sucrose test in an attempt to avoid bias associated with the assumption that gastroscopy is a perfect test.

## Abbreviations

AUC: area under the curve
Bayesian LC: Bayesian latent class
BW: body weight
CSL: clinically significant lesion
EGUS: equine gastric ulcer syndrome
GC–FID: gas chromatography–flame ionization detection
GDL: glandular lesion
GL: gastric lesion
PI: probability interval
ROC: receiver operator characteristics
SD: standard deviation
Se: sensitivity
Sp: specificity
SQL: squamous lesion
95% CI: 95% confidence intervals
